# *Lactococcus petauri* LZys1 modulates gut microbiota, diminishes ileal FXR-FGF15 signaling, and regulates hepatic function

**DOI:** 10.1128/spectrum.01716-24

**Published:** 2025-04-17

**Authors:** Ouyang Li, Yingshun Zhou, Dayoung Kim, Han Xu, Zhijun Bao, Fan Yang

**Affiliations:** 1 Shanghai Key Laboratory of Clinical Geriatric Medicine, Shanghai, China; 2 Digestive Endoscopy Center, Huadong Hospital, Fudan University 159396, Shanghai, China; 3 Department of Pathogenic Biology, Public Center of Experimental Technology of Pathogen Biology Technology Platform, Southwest Medical University, Luzhou, Sichuan, China; 4 Department of Gerontology, Huadong Hospital, Fudan University 159396, Shanghai, China; Lerner Research Institute, Cleveland, Ohio, USA

**Keywords:** *Lactococcus petauri *LZys1, gut microbiota, FGF15, liver function, FXR

## Abstract

Recent studies have indicated that *Lactococcus petauri* LZys1 (*L. petauri* LZys1), isolated from healthy human feces, exhibits a promising probiotic profile *in vitro*. However, its impact on the physiological status of the host *in vivo* remains uncertain. The objective of our study was to investigate the effects and mechanisms of orally administering *L. petauri* LZys1 on gut microbiota and liver function in mice. We administered *L. petauri* LZys1 through daily oral gavage to C57BL/6 male mice. Subsequently, we analyzed changes in gut microbiota composition using 16S rRNA sequencing and quantified alterations in hepatic-intestinal bile acid (BA) profile. Serum biochemical parameters were assessed to evaluate liver function. Our findings revealed that *L. petauri* LZys1 led to an increase in body weight, liver mass, and serum aminotransferase levels. Oral administration altered the gut microbiota composition, resulting in reduced diversity and abundance of intestinal bacteria. Additionally, the profiles of BAs were suppressed across organs, associated with the downregulation of the ileum’s farnesoid X receptor (FXR)/fibroblast growth factor 15 (FGF15) signaling pathway. The decrease in circulating FGF15 mediated the downregulation of hepatic fibroblast growth factor receptor 4 (FGFR4)/FXR, disrupting BA metabolism and fatty acid oxidation. Our findings suggest that *L. petauri* LZys1 may impact liver function by influencing the gut microbiota-mediated ileal FXR–FGF15 axis and inhibiting hepatic bile acid metabolism.

**IMPORTANCE:**

This work elucidated the impact of *L. petauri* LZys1 on host gut microbiota metabolism and hepatic physiological metabolism. We observed that *L. petauri* LZys1 administration induced liver weight gain and biochemical parameters changes, in addition to a altered gut microbiota and suppressed bile acid (BA) profiles. Furthermore, we propose that changes in liver status are related to the enterohepatic farnesoid X receptor–fibroblast growth factor axis, which alters bile acid metabolism and disrupts liver function. The above findings suggest that attention should be paid to the effect of probiotics on liver function.

## INTRODUCTION

In recent years, the view that gut microbiota is an invisible organ of the human body has become mainstream. It carries the largest pool of genetic material in the body composed of approximately 10^13^–10^14^ bacteria, even larger than the human genome, vastly expanding the metabolic capacity of the body (1). Bile acids (BAs), synthesized via oxidation of cholesterol in hepatocytes, are one of the most important gut microbiota metabolites. The classical pathway produces the primary BAs cholic acid (CA) and chenodeoxycholic acid (CDCA) in humans, or muricholic acid (MCA) in mice, via cholesterol 7α-hydroxylase (CYP7A1), sterol 12α-hydroxylase (CYP8B1), and cholesterol 27-hydroxylase (CYP27A1). The CYP7A1 is the only rate-limiting enzyme in BA synthesis and is responsible for CA and CDCA. The microsomal CYP8B1 is required for synthesis of CA; without CYP8B1, the product is CDCA. The alternative pathway generates CDCA through CYP27A1 and oxysterol 7α-hydroxylase (CYP7B1), by hydroxylating the cholesterol side chain (2–4). CA and CDCA are unconjugated primary BAs, stored in gallbladder, forming conjugated primary BAs—taurocholic acid (TCA), taurochenodeoxycholic acid (TCDCA), and tauromuricholate (TMCA)—by binding to either glycine or taurine through bile acyl­-CoA synthetase (BACS) and bile acid­-CoA:amino acid N­-acyltransferase (BAAT) (5, 6). Following the excretion from the hepatocytes to bile canaliculus, depending on the bile salt export pump (BSEP) and multidrug resistance­-associated protein 2 (MRP2), the primary BAs flow into the intestine through major duodenal papilla (7, 8). Alternatively, other translocators like MRP3, MRP4, OSTα, and OSTβ existing in hepatocytes also excrete BAs into systemic circulation (9). The secondary BAs are transformed by gut microbiota in the digestive cavity from primary BAs. Firstly, bile salt hydrolases (BSHs) are responsible for the deconjugation to glyco-conjugated and tauro-conjugated CA and CDCA, produced by a vast number of bacteria, including but not limited to *Bacteroides*, *Clostridium*, *Lactobacillus*, *Bifidobacterium,* and *Listeria* (10). Secondly, deoxycholic acid (DCA) and lithocholic acid (LCA) are formed by 7α-dehydroxylation by *Bacteroides*, *Eubacterium*, *Clostridium*, *Escherichia*, *Eggerthella*, *Peptostreptococcus*, and *Ruminococcus* (11). Thirdly, in addition to the above bacteria, more microbes like *Eggerthella* and *Peptostreptococcus* are also involved in the oxidation and epimerization of hydroxyl groups at C7, generating βMCA and αMCA (12, 13). The BA metabolism by gut microbiota is a huge enzymatic reaction network, which ultimately promotes the reabsorption of BAs via apical sodium-dependent BA transporter (ASBT) in the terminal jejunum and ileum (14, 15). Enterocytes secrete BAs into the bloodstream through the basolateral BA transporters OSTα, OSTβ, and MRP2 (9). BAs are taken back to the liver along with portal circulation, completing the enterohepatic circulation of BAs.

Farnesoid X receptor (FXR) belongs to the nuclear receptor family and is highly expressed in the gastrointestinal tract and liver as a key sensor to mediate BA feedback inhibition of BA synthesis, facilitate BA transportation among hepatocytes, intestinal cavity, and enterocyte, and modulate BA metabolism from primary to secondary BAs, preventing liver injury and cholestasis. BAs activate FXR, and activation of FXR in the liver generates the small heterodimer partner (SHP) to hamper transcription of the CYP7A1 gene in hepatocytes (16, 17). Additionally, fibroblast growth factor 15 (FGF15)/FGF19 (FGF15 in mice, FGF19 in humans) produced in the intestine is an FXR-regulated hormone, activating the compounds of FGFR4/β-klotho (KLB) and also inhibiting CYP7A1 and CYP8B1 gene transcription (18, 19). BSEP, MRP2, OSTα, and OSTβ are directly or indirectly induced by FXR, while ASBT is inhibited, making FXR a core factor to maintain low intrahepatic BA concentration and to prevent cholestatic liver injury (19). As for downstream pathways, FXR serves as a central regulator of cholesterol and lipid homeostasis by modulating cholesterol breakdown and distribution. Peroxisome proliferator-activated receptor α (PPARα) as a main ligand of FXR is involved in mitochondrial fatty acid uptake and β-oxidation, promoting lipolysis, while inhibiting lipogenesis (20). Some reports also proved that the activation of FXR by CDCA induced PPARα and improved its downstream recruitment of the mitochondrial fatty acid transporter carnitine palmitoyltransferase-1α (CPT-1α) (21). In addition to regulating BA and lipid metabolism, activation of FXR has anti-inflammatory effects. FXR directly transrepresses nuclear factor-κB (NF-κB) activation in the liver and decreases classical pro-inflammatory cytokines such as tumor necrosis factor α (TNFα), interleukin-6 (IL-6), and interleukin-1β (IL-1β) (22, 23).

*L. petauri* LZys1 is a type of lactic acid bacteria, which was first isolated from healthy male gut. Laboratory studies have demonstrated that *L. petauri* LZys1 can resist acid and protect against pathogenic bacteria like *Salmonella*, *Escherichia coli,* and *Klebsiella pneumoniae* (24). It was originally intended to be used as a probiotic for the treatment of digestive diseases, but the lack of *in vivo* experiments cannot confirm the safety and medicinal value of *L. petauri* LZys1. In this study, we investigated the impact of *L. petauri* LZys1 on liver function, focusing on the regulation of BA metabolism and alteration of gut microbiota composition.

## MATERIALS AND METHODS

### Strain culture

*L. petauri* LZys1 was obtained from the laboratory of pathogenic biology in Southwest Medical University and cultured in MRS (de Man, Rogosa and Sharpe) medium at 37°C. The *L. petauri* LZys1 was collected after repeated washing and centrifugation with ice-cold phosphate-buffered saline (PBS). Then, the live bacteria were resuspended in sterile PBS to reach a concentration of 2.5 × 10^9^ CFU/mL. The bacteria were re-sequenced before use.

### Animal treatment

Six-week-old specific pathogen-free (SPF) male mice (C57BL/6) were obtained from the Shanghai Sipeifu Laboratory Animal Care. Mice were housed four per cage with a 12 h light/dark schedule in a temperature- and humidity-controlled vivarium within an SPF environment, with *ad libitum* access to food and water. After 2 weeks of adaptive feeding, all the mice were divided into two groups randomly (the Lzys1 group and the control group). Mice in the LZys1 group were treated with a daily oral gavage with 0.2 mL of live *L. petauri* LZys1 suspension (approximately 0.5 × 10^9^ CFU total counts), while mice in the control groups were administered PBS instead. After 8 weeks, the mice were euthanized, and blood, liver, ileum, cecum, and fecal samples were collected and immediately stored at −80°C.

### Oral glucose tolerance test (OGTT)

After 16 h of fasting, mice were weighed, and their baseline blood glucose levels were measured using a glucometer (Accu-Chek Instant, Roche). The mice were administered oral gavage of 20% glucose at the dosage of 2 g/kg body weight, and their blood glucose levels were measured at 15, 30, 60, and 120 min. The area under the glucose tolerance test curve was calculated using GraphPad Prism 9.

### Body composition

After 16 h fasting (from 8:00 a.m. to 12:00 a.m.), mice body composition (fat and lean mass) was analyzed by animal body composition analyzer (EchoMRI).

### Serum biochemical analysis

Serum levels of liver function and blood fat including alanine aminotransferase (ALT), aspartate transaminase (AST), alkaline phosphatase (ALP), cholinesterase, albumin (ALB), cholesterol, triglyceride, and high-density lipoprotein (HDL) cholesterol were determined by ADVIA Chemistry XPT automatic analyzer. Serum FGF15 was detected using an ELISA kit (X-Y biotechnology, XY-FGF15-Mu) according to the manufacturer’s instructions.

### 16S rRNA sequencing and bioinformatics analysis

Fecal pellets were promptly flash-frozen in liquid nitrogen upon collection and stored at −80°C. A 1–2 g fecal sample was used for each mouse. DNA extraction from the fecal samples was conducted, and the DNA was quantified using Nanodrop. Universal primers with specific barcodes targeting the conserved V3-V4 regions of 16S rDNA were used (341F:5′-CCTAYGGGRBGCASCAG-3′, 806R: 5′-GGACTACNNGGGTATCTAAT-3′). The high-fidelity PCR products were subjected to fluorescence quantification using the Quant-iT PicoGreen dsDNA Assay Kit as the fluorescence reagent and a microplate reader (BioTek, FLx800) as the quantification instrument. The sequencing libraries were prepared using the TruSeq Nano DNA LT Library Prep Kit from Illumina. The library quality was assessed using the Agilent Bioanalyzer with the Agilent High Sensitivity DNA Kit. Sequencing was performed using the NovaSeq 6000 SP Reagent Kit (500 cycles) from Illumina. Single-ended reads (400 bp/600 bp) were acquired and processed to obtain clean reads. The raw data were quality controlled (QC) using fastp software (https://github.com/OpenGene/fastp, version 0.20.0), and were spliced using FLASH software (http://www.cbcb.umd.edu/software/flash, version 1.2.11). Based on default parameters, optimized sequences after QC splicing were noise reduced using the DADA2 (25) plugin in the QIIME 2 process (26). Sequences that have a similarity of higher than 97% were clustered into an amplicon sequence variant (ASV). After removal of all samples annotated to chloroplast and mitochondrial sequences, representative ASVs were extracted and annotated with Mothur based on the Silva database (v.138) and were analyzed for species taxonomy using the Naive Bayes classifier in QIIME 2. The α-diversity and β-diversity were calculated by Majorbio online software. Principal coordinate analysis (PCoA) based on UniFrac distance was conducted to compare the community distributions, and the significance was analyzed by Majorbio online software. The Wilcoxon rank-sum test or *t*-test was adopted for intergroup analysis. The community constitution at various taxonomic levels (phyla, families, genera, species) was analyzed by MetaStat.

### BA profile analysis

Ileum tissues, cecal content , and hepatic tissues were used to quantify BAs. Approximately 10 mg of tissue was weighed, and 20 mg of prechilled zirconium oxide beads and 15 µL of ultrapure water were added. A prechilled acetonitrile and methanol mixture was added (80:20, vol/vol). After centrifugation at 13,500 *g* and 4°C for 20 min, 40 µL of supernatant was carefully collected and gently mixed with 150 µL of acetonitrile and methanol mixture solution (50:50, vol/vol). Five microliters of supernatant was extracted for later detection after centrifugation at 13,500 *g* and 4°C for 20 min. The ultra-high-performance liquid chromatography tandem mass spectrometry (UPLC-MS/MS, ACQUITY UPLC-Xevo TQ-S, Waters Corp., Milford, MA, USA) was used to quantify quality control samples and supernatant above. The parameter of UPLC-MS/MS is shown in Supplementary 1. The MS system (Waters Corp) was used for BA quantification following previous protocols (27). MassLynx software (version 4.1, Waters, Milford, MA, USA) scored each BA, created a standard curve, and quantified the process. Mass spectrometry-based quantitative metabolomics obtains actual BA concentrations by comparing metabolites in samples of unknown concentration with a set of standard samples of known concentration. The BAs profile were permformed by Metabo-Profile Biotechnology. The Spearman correlation heatmap was drawn by Majorbio online software.

### RNA extraction and quantitative real-time PCR

Total RNA of liver and ileum was isolated by Trizol reagent (bioTNT, Shanghai, China) and treated with RNase-free DNase (Takara Bio Inc., Kusatsu, Shiga, Japan). Reverse transcription was performed with TaKaRa RNA PCR kit (Takara Bio Inc., Kusatsu, Shiga, Japan). The cDNA of targeted genes was amplified by using SYBR Green (Takara Bio Inc., Kusatsu, Shiga, Japan) in StepOne (Applied Biosystems). Samples were assayed in triplicate, and β-actin was used as an internal control. All primer sequences used were shown in Table 1.

**TABLE 1:** Primer sequences in this study

Gene symbol	Gene name		Primer sequence (5´ to 3´)
β-Actin	Actin, beta	F	GGCTGTATTCCCCTCCATCG
		R	CCAGTTGGTAACAATGCCATGT
Nr1h4	Nuclear receptor subfamily 1, group H, member 4	F	ACAGCGAAGGGCGTGACTTG
		R	GGTCTGTTGGTCTGCCGTGAG
Fgf15	Fibroblast growth factor 15	F	TCCGCTGGTCCCTATGTCTCC
		R	CGTCCTTGATGGCAATCGTCTTC
Nr0b2	Nuclear receptor subfamily 0, group B, member 2	F	CGGCTGCTAGAGTGCTGCTG
		R	GTACCGCTGCTGGCTTCCTC
Slc10a2	Solute carrier family 10, member 2	F	CACTTGCTCCACACTGCTTGC
		R	TTCCCGAGTCAACCCACATCTTG
Fabp6	Fatty acid binding protein 6	F	GCAGGACGGACAGGACTTCAC
		R	CCTCCATCTTCACGGTAGCCTTG
Slc51a	Solute carrier family 51, member A	F	GCTTGCTCACCTCCCTACTCTTC
		R	TACCCAACCTTGTCGTCTTTCCTTC
Slc51b	SLC51 subunit beta	F	TCCTGGCAGTCCTGGTGGTC
		R	TTTGTCTTGTGGCTGCTTCTTTCG
Klb	Klotho beta	F	GAACAGAAGTCCTGCGTGTGGTC
		R	AGCAGCAACCGAAGAAGATGAGTG
Fgfr4	Fibroblast growth factor receptor 4	F	ACTCTCGCCAGCCTGTCACTATAC
		R	AGACGGACACCTCGCACCAG
Cyp7a1	Cytochrome P450 family 7, subfamily A, member 1	F	AGCAGCCTCTGAAGAAGTGAATGG
		R	AGAGCCGCAGAGCCTCCTTG
Pparα	Peroxisome proliferator-activated receptor alpha	F	CTTCACGATGCTGTCCTCCTTGATG
		R	GATGTCACAGAACGGCTTCCTCAG
Ppargc1a	PPARγ coactivator 1 alpha	F	GTGCCACCGCCAACCAAGAG
		R	TTCCTCGTGTCCTCGGCTGAG
Cpt1a	Carnitine palmitoyltransferase 1A	F	CTCAAGATGGCAGAGGCTCA
		R	GGGGAACACACCAGTGATGA
Abcb11	ATP-binding cassette, subfamily B, member 11	F	CCTGCTTCTGGACATGGCTACC
		R	ATAGGCGATGGGCAACTGAGATG
Abcc3	ATP-binding cassette, subfamily C, member 3	F	GAGGAGATAGCAGAGACAGGCAATG
		R	GGCAGATAGATAGCGTGGTACAGAG
Abcc4	ATP-binding cassette, subfamily C, member 4	F	GACGCCGACATCTACCTCCTTG
		R	ACGCCTGACAGATACACAGTTGG
Tgfb1	Transforming growth factor beta 1	F	ACCGCAACAACGCCATCTATGAG
		R	GGCACTGCTTCCCGAATGTCTG
Acta2	Actin alpha 2, smooth muscle, aorta	F	CATCAGGGAGTAATGGTTGGAATGGG
		R	GTGTTCTATCGGATACTTCAGCGTCAG
Mmp2	Matrix metallopeptidase 2	F	ACCATGCGGAAGCCAAGATGTG
		R	AGGGTCCAGGTCAGGTGTGTAAC
Mmp3	Matrix metallopeptidase 3	F	GACGATGATGAACGATGGACAGAGG
		R	TGTGGAGGACTTGTAGACTGGGTAC
Timp1	TIMP metallopeptidase inhibitor 1	F	AGGATTCAAGGCTGTGGGAAATGC
		R	CTTCACTGCGGTTCTGGGACTTG
Timp2	TIMP metallopeptidase inhibitor 2	F	CGCTTAGCATCACCCAGAAGAAGAG
		R	AGTCCATCCAGAGGCACTCATCC
Lgals3	Galectin 3	F	AGACAGCTTTTCGCTTAACGA
		R	GGGTAGGCACTAGGAGGAGC
Il6	Interleukin 6	F	TAGTCCTTCCTACCCCAATTTCC
		R	TTGGTCCTTAGCCACTCCTTC
Il1b	Interleukin 1 beta	F	GCAACTGTTCCTGAACTCAACT
		R	ATCTTTTGGGGTCCGTCAACT
Slc27a5	Solute carrier family 27 (fatty acid transporter), member 5	F	AGAGCCTCTGAGGGACAAACAGG
		R	GCCCAGGAAGGGTTGGTTCTTTC
Baat	Bile acid-coenzyme A:amino acid N-acyltransferase	F	CCTTTCAGGTGGTGTGCCTTCAG
		R	ACCCACTTCACTGGCCCTGTAG

### Western blot

Total protein from liver was extracted using RIPA buffer containing 50 mM Tris (pH 7.4), 150 mM NaCl, 1% Triton X-100, 1% sodium deoxycholate, 0.1% SDS, 1% phosphatase inhibitor, and protease inhibitor. The proteins were separated by electrophoresis on 10% to 15% SDS-polyacrylamide gels and transferred moist to polyvinylidene difluoride membranes. The membranes were blocked with 5% defatted milk in Tris-buffered saline with Tween 20 (TBST) at room temperature for 2 h and then incubated with FXR (Santa Cruz Biotechnology, sc-25309, 1:100), FGFR4 (Santa Cruz Biotechnology, sc-136988, 1:100), and β-actin (CST, 12262, 1:100). After washing, the membranes were incubated with the secondary antibody (CST, 7074, 1:6,000) for 2 h. The membranes were exposed to enhanced chemiluminescence (ECL) buffer after another five washes, and the blots were detected by the ChemiDoc XRS+ System (BIO-RAD, Berkeley, USA).

### Statistical analysis

All statistical analyses were performed using GraphPad Prism 9.0 (GraphPad, La Jolla, CA, USA). The results were expressed as the means  ± standard deviations or means  ± standard error of the mean (SEM). The statistical significance of the differences between the two experimental groups was evaluated using a two-sample *t*-test or Wilcoxon rank-sum test. A value of *P* < 0.05 was considered statistically significant.

## RESULTS

### *L. petauri* LZys1 regulates liver function

**Fig 1:**
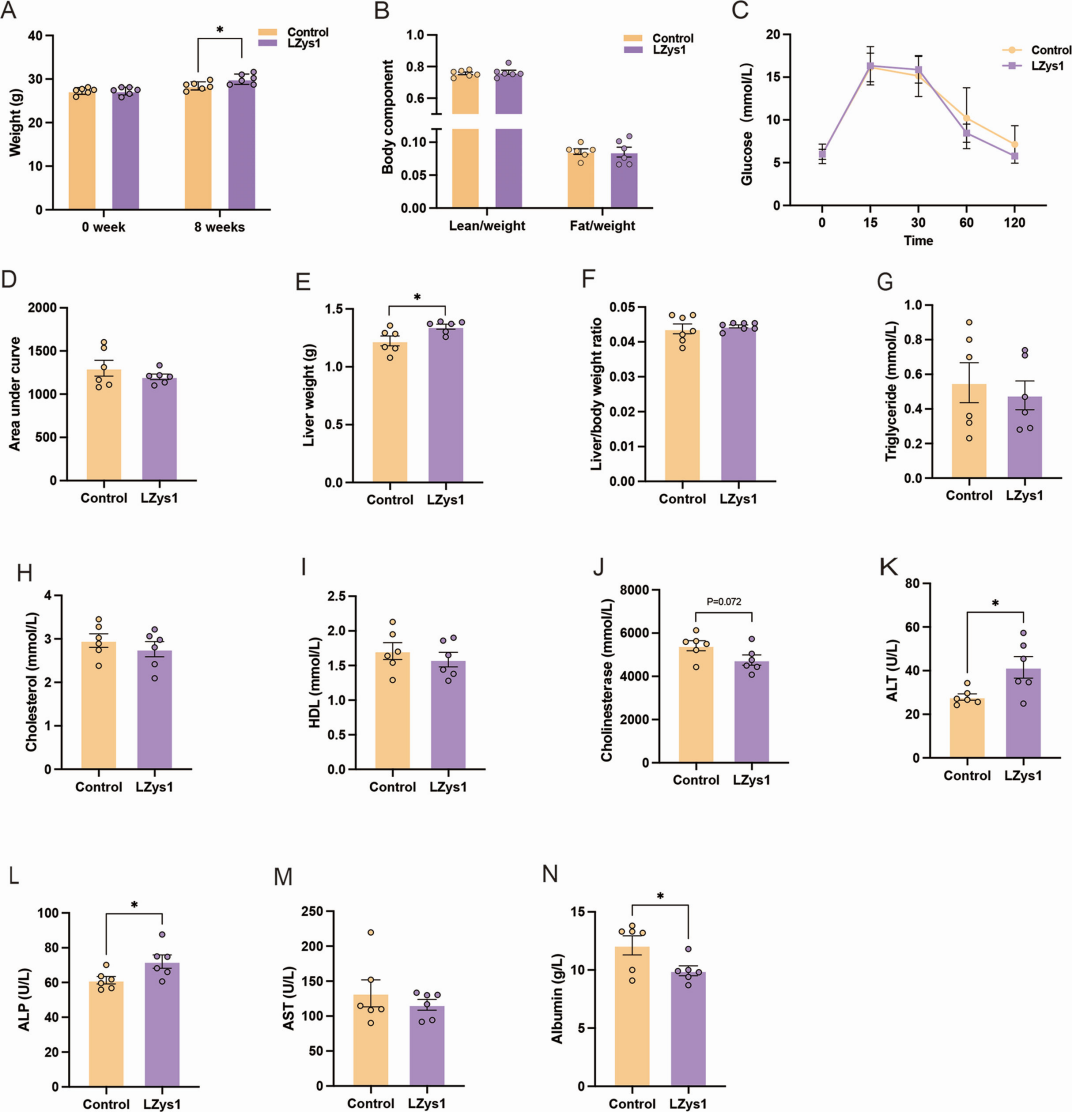
*L. petauri* LZys1 regulates liver function. (**A**) The weight changes at the start (0 week) and end (8 weeks) of the *L. petauri* LZys1 administration. (**B**) Body composition analysis after 8 weeks. The OGTT curves (**C**) and their area under the curves (**D**) are presented for both the LZys1 group and the control group. (**E**) The liver mass of the LZys1 group and the control group after 8 weeks. (**F**) The ratio of liver mass to body weight. Serum biochemical indicators including triglyceride (**G**), cholesterol (**H**), HDL (**I**), cholinesterase (**J**), ALT (**K**), ALP (**L**), AST (**M**), and ALB (**N**). Data (**A, B, D–N**) are expressed as mean ± SEM and were analyzed using two-sample *t*-test, *P < 0.05. Number of samples is six per group.

After 8 weeks of *L*. *petauri* LZys1 administration, we found that Lzys1 mice gained more body weight (Fig. 1A), while the body composition (Fig. 1B) showed no significant difference. The OGTT results indicated that systemic glucose anabolism remains stable (Fig. 1C and D). Interestingly, we observed more liver weight (Fig. 1E) in the LZys1 group, and the mass ratio of liver and the whole body showed no significant difference (Fig. 1F), indicating that *L. petauri* LZys1 induced greater liver mass than general weight. Then, we detected the serum biochemical indices. We found a significant increase in serum ALT (Fig. 1K) and ALP (Fig. 1L), and an obvious decrease in cholinesterase (Fig. 1J) and ALB (Fig. 1N); however, the AST, cholesterol, triglyceride, and HDL were found to have no statistical significant changes between the groups (Fig. 1G through I and M).

### *L. petauri* LZys1 induces the dysbiosis of the gut microbiota

**Fig 2:**
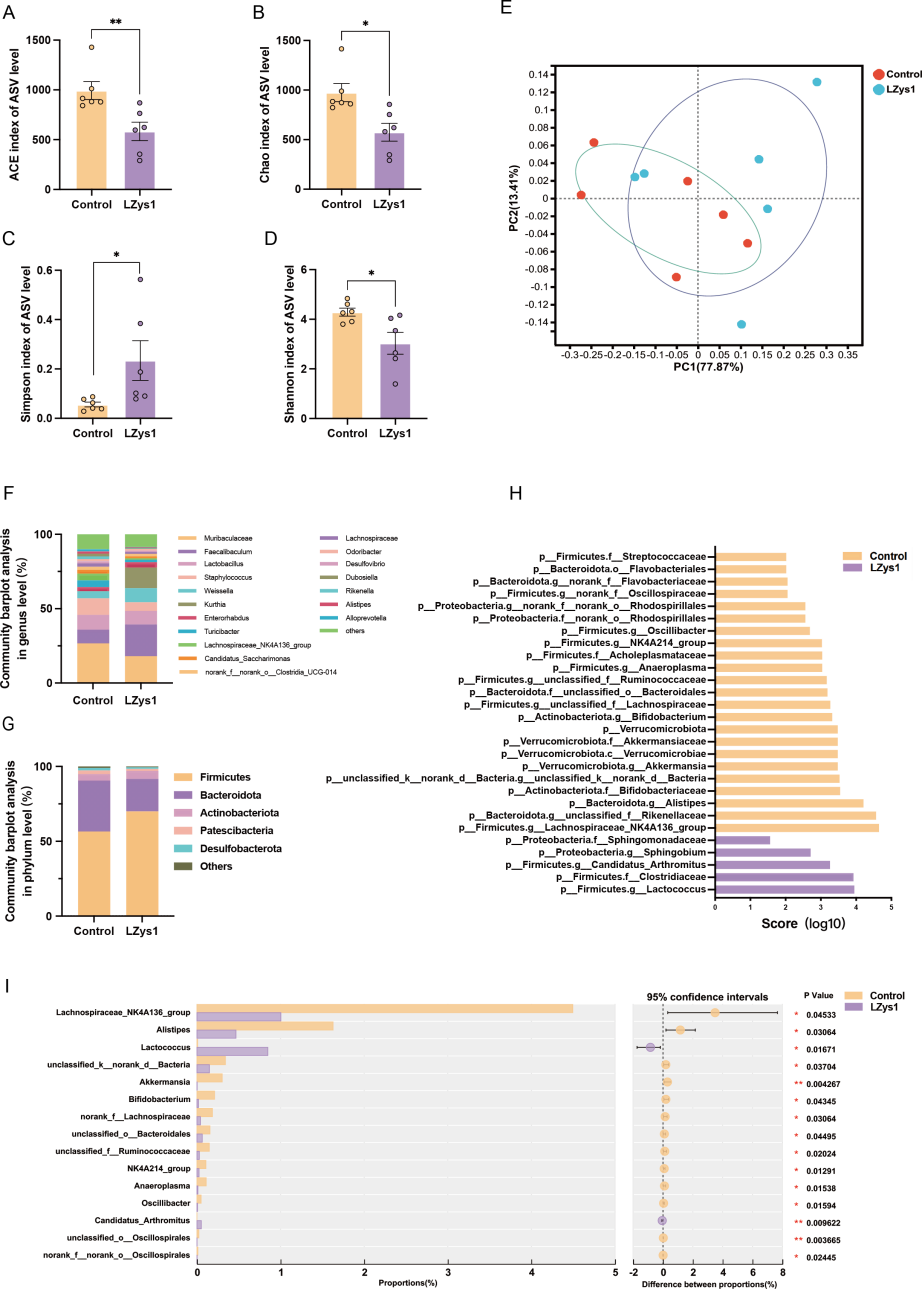
*L. petauri* LZys1 induces the dysbiosis of the gut microbiota. The Ace index (**A**), Chao index (**B**), Simpson index (**C**), and Shannon index (**D**) of the gut microbiota in LZys1 and control mice. (**E**) Principal coordinates analysis on the basis of Bray-Curtis dissimilarity of the LZys1 and control groups. (**F**) Bacterial composition at the genus level. (**G**) Bacterial composition at the phylum level. (**H**) The LEfSe analysis between the LZys1 and control groups. (**I**) The differences between groups at the genus level using the Wilcoxon rank-sum test. The 95% CIs were calculated by the method of bootstrap. Data (A, B, C, D, F, G) are expressed as mean ± SEM and compared by two-sample *t*-test, *P < 0.05; **P < 0.01. Number of samples is six per group.

To find out the alteration of the gut microbiota, we examine fecal microbial communities using 16S rRNA sequencing analysis. The Chao, Simpson, Shannon, and Ace index were used to describe the α-diversity of the gut microbiota (Fig. 2A through D). Though the Simpson index showed an increase in α-diversity, other models like Ace, Chao, and Shannon indices suggested the α-diversity decreased in the LZys1 group. In contrast to the control group, *L. petauri* LZys1 administration reduced the community richness and diversity of gut microbiota. PCoA clustering was was utilized to differentiate the construction samples using unweighted UniFrac distances (Fig. 2E). The β-diversity showed no difference between the LZys1 and the control group. At the genus level, the Lzys1 group showed an obvious increase in the abundance of *Faecalibaculum*, *Weissella*, and *Kurthia*, while the community of *Muribaculaceae* reduced drastically (Fig. 2F). The Lzys1 group showed increased abundance of *Firmicute*s and decreased abundance of *Bacteroidetes* (Fig. 2G) at the phylum level. The linear discriminant analysis effect size (LEfSe) analysis also showed a significant decrease in 23 bacteria, including *p__Firmicutes.g__Ruminococcus*, *p__Actinobacteriota.g__Bifidobacterium*, *p__Bacteroidota.g__Bacteroides*, *p__Firmicutes.f__Clostridium*, and *p__Firmicutes.g__Lachnospiraceae* in the LZys1 group (Fig. 2H), indicating the dysbiosis of the gut microbiota. Using the Wilcoxon rank-sum test, we identified the genus of the significant diversity between the LZys1 and control groups. The *p__Firmicutes.g__Lachnospiraceae_NK4A136_group*, *p__Bacteroidota.g__Alistipes*, *p__Verrucomicrobiota.g__Akkermansia*, and *p__Actinobacteriota.g__Bifidobacterium* exhibited a large decrease in the LZys1 group (Fig. 2I).

### *L. petauri* LZys1 disturbs BA metabolism

**Fig 3:**
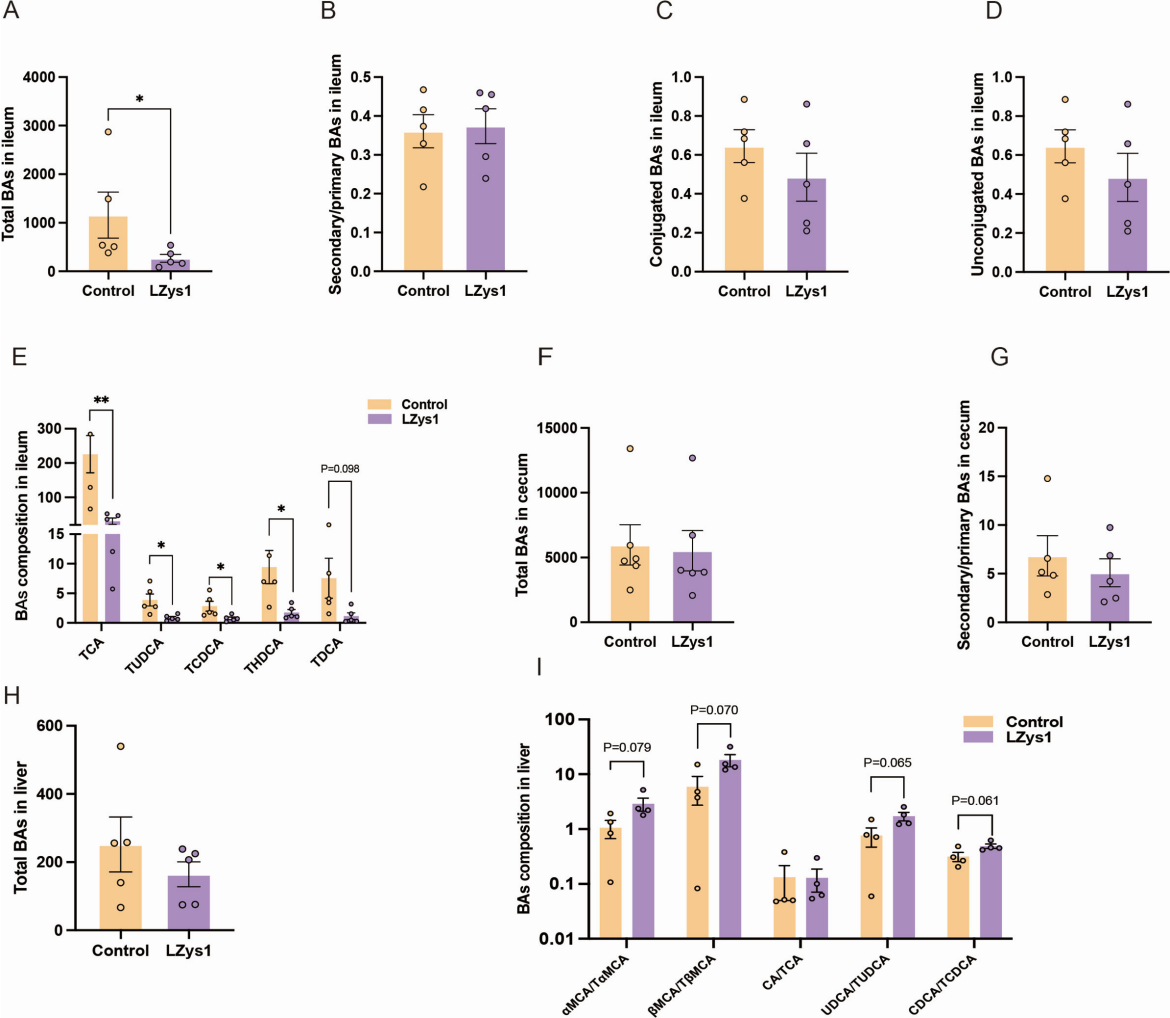
*L. petauri* LZys1 alters BA metabolism. (**A**) The total amount of BAs in the ileum of the LZys1 and control groups. (**B**) The ratio of secondary BAs and primary BAs in the ileum. (**C**) The amount of the conjugated BAs in the ileum. (**D**) The amount of the unconjugated BAs in the ileum. (**E**) The significantly different BA components between the LZys1 and control groups in the ileum. (**F**) The total amount of BAs in cecal contents of the LZys1 and control groups. (**G**) The ratio of secondary BAs and primary BAs in cecal contents. (**H**) The total amount of BAs in the liver. (**I**) The ratio of unconjugated BAs and conjugated BAs in the liver. Data (**A–I**) are expressed as mean ± SEM and compared by two-sample *t*-test; *P 0.05, **P < 0.01. Number of samples is five per group.

Considering the above changes in the LZys1 group, we further detected the BA profiles in ileum tissues, cecal contents, and hepatic tissues. As we can see, the total disturbance of BAs in the ileum was significantly lower in the LZys1 group than in the control (Fig. 3A). The ratio of secondary BAs and primary BAs in the ileum exhibited no statistical differences between the two groups (Fig. 3B). Following the administration of *L. petauri* LZys1, there was a slight decrease in the levels of both conjugated and unconjugated BAs (Fig. 3C and D), indicating that the contents of BAs in the lumen of the intestine were reduced, or the reabsorption of BAs in the epithelial cells of the ileum was restrained. In more detail, TCA, tauroursodeoxycholic acid (TUDCA), TCDCA, taurohyodeoxycholic acid (THDCA), and taurodeoxycholate (TDCA) were reduced significantly in the Lzys1 group (Fig. 3E). The drastic reduction of TCA, a strong activator of FXR that promotes ileum BA transportation, suggested the suppression of BA metabolism in the enterohepatic circulation. Furthermore, the total amount of BAs and the ratio of secondary BAs and primary BAs in cecal contents exhibited no significant changes (Fig. 3F and G). Although the total BA content of the liver did not decrease dramatically (Fig. 3H), the ratio of αMCA to TαMCA, βMCA to TβMCA, UDCA (ursodeoxycholic acid) to TUDCA, and CDCA to TCDCA decreased relatively (Fig. 3I), suggesting the composition of conjugated BAs is reduced relative to total BAs.

### Association of altered gut microbiota and BAs

**Fig 4:**
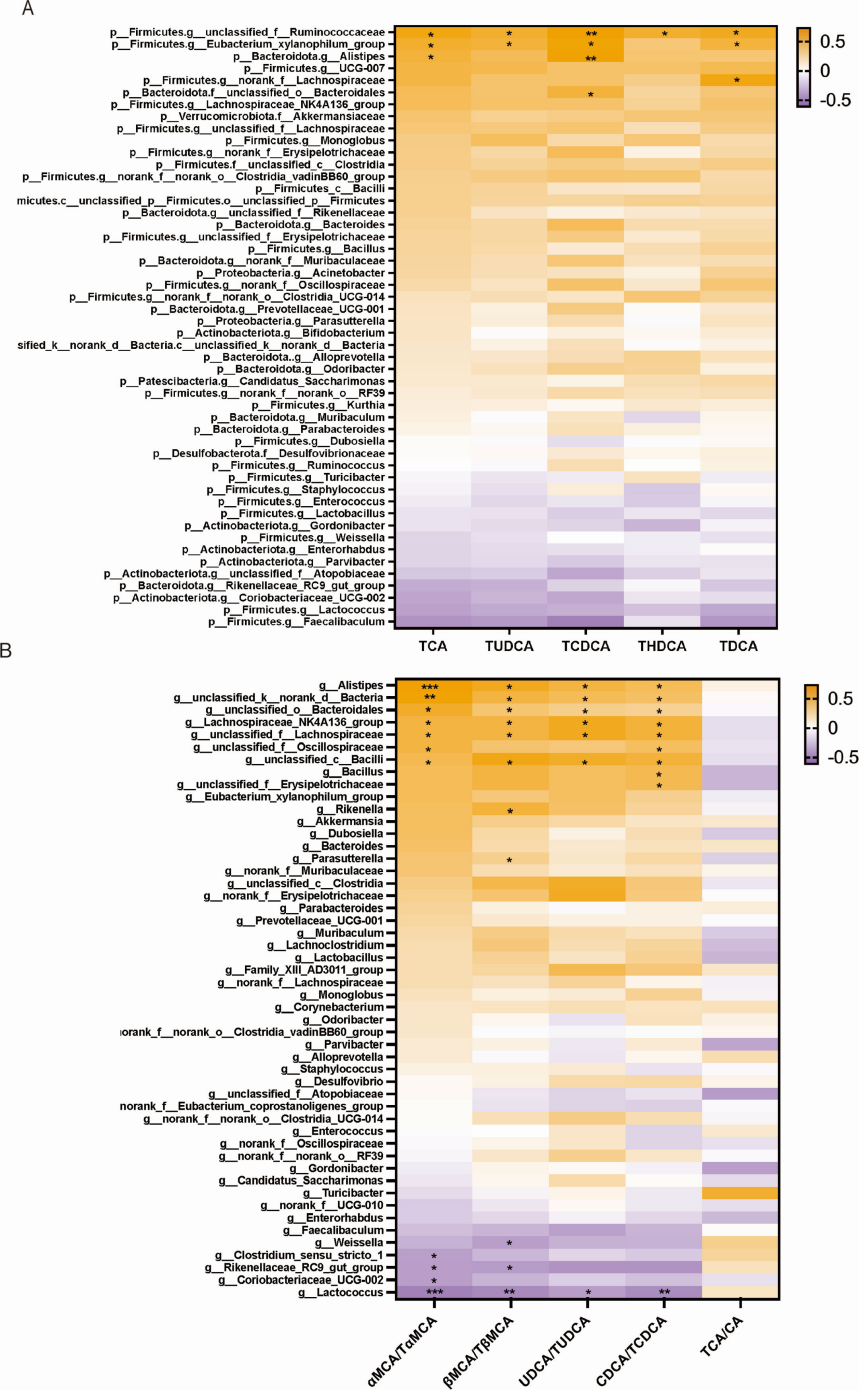
Alteration in gut microbiota associates with BA metabolism. (A) Heatmap exhibiting Spearman correlation coefficient between gut microbiota and different BA components of the LZys1 and control groups in the ileum. (B) Heatmap exhibiting Spearman correlation coefficient between gut microbiota and the ratio of unconjugated BAs and conjugated BAs in the liver. *P < 0.05, **P < 0.01. The number of samples is five per group.

As previously mentioned, primary BAs are modified and transformed into secondary BAs by gut microbiota, as well as the deconjugation of conjugated BAs. Thus, the correlation between intestinal microbiota and BAs in the ileum (Fig. 4A) was analyzed. Multiple genera were significantly correlated with BAs. In the ileum, *p__Firmicutes.g__Eubacterium* was associated with TCA, TUDCA, TCDCA, and TDCA; *p__Firmicutes.g__Ruminococcus* had significant correlation with TCA, TUDCA, TCDCA, THDCA, and TDCA; *p__Bacteroidota.g__Alistipes* was responsible for the changes of TCA and TCDCA; and *p__Firmicutes.g__norank_f__Lachnospiraceae* was associated with TDCA. As for the transform of hepatic BAs, we performed another Spearman correlation heatmap between gut microbiota and the percentage of conjugated to unconjugated BAs in liver (Fig. 4B). The ratio of αMCA to TαMCA had positive correlation with *p__Bacteroidota.g__Alistipes*, *p__Firmicutes.f__Lachnospiraceaee, p__Firmicutes_c__Bacilli, p__Firmicutes. _f__Oscillospiraceae, p__Firmicutes.g__Bacillus*, and *p__Bacteroidota.o__Bacteroidales,* and negative correlation with *p__Actinobacteriota.g__Coriobacteriaceae, p__Bacteroidota.g__Rikenellaceae,* and *p__Firmicutes.f__Clostridium*. Similarly, *p__Bacteroidota.g__Alistipes*, *p__Firmicutes.f__Lachnospiraceae, p__Firmicutes_c__Bacilli, p__Bacteroidota.o__Bacteroidales, p__Bacteroidota.g__Rikenellaceae*, and *Parasuterea* were positively related to the ratio of βMCA to TβMCA, while *p__Bacteroidota.g__Rikenellaceae* and *p__Firmicutes.g__Weissella* were negatively related. The rate of UDCA to TUDCA was positively related to *p__Bacteroidota.g__Alistipes, p__Firmicutes.f__Lachnospiraceae, p__Firmicutes_c__Bacilli,* and *p__Bacteroidota.o__Bacteroidales*. Likewise, there was a positive relationship between the ratio of CDCA to TCDCA and *p__Bacteroidota.g__Alistipes, p__Firmicutes.f__Lachnospiraceae, p__Firmicutes. _f__Oscillospiraceae, p__Firmicutes_c__Bacilli, p__Bacteroidota. o__Bacteroidales*, and *p__Firmicutes.g__unclassified_f__Erysipelotrichaceae*.

### *L. petauri* LZys1 downregulates the FXR/FGF15 pathway and disturbs liver metabolism

**Fig 5:**
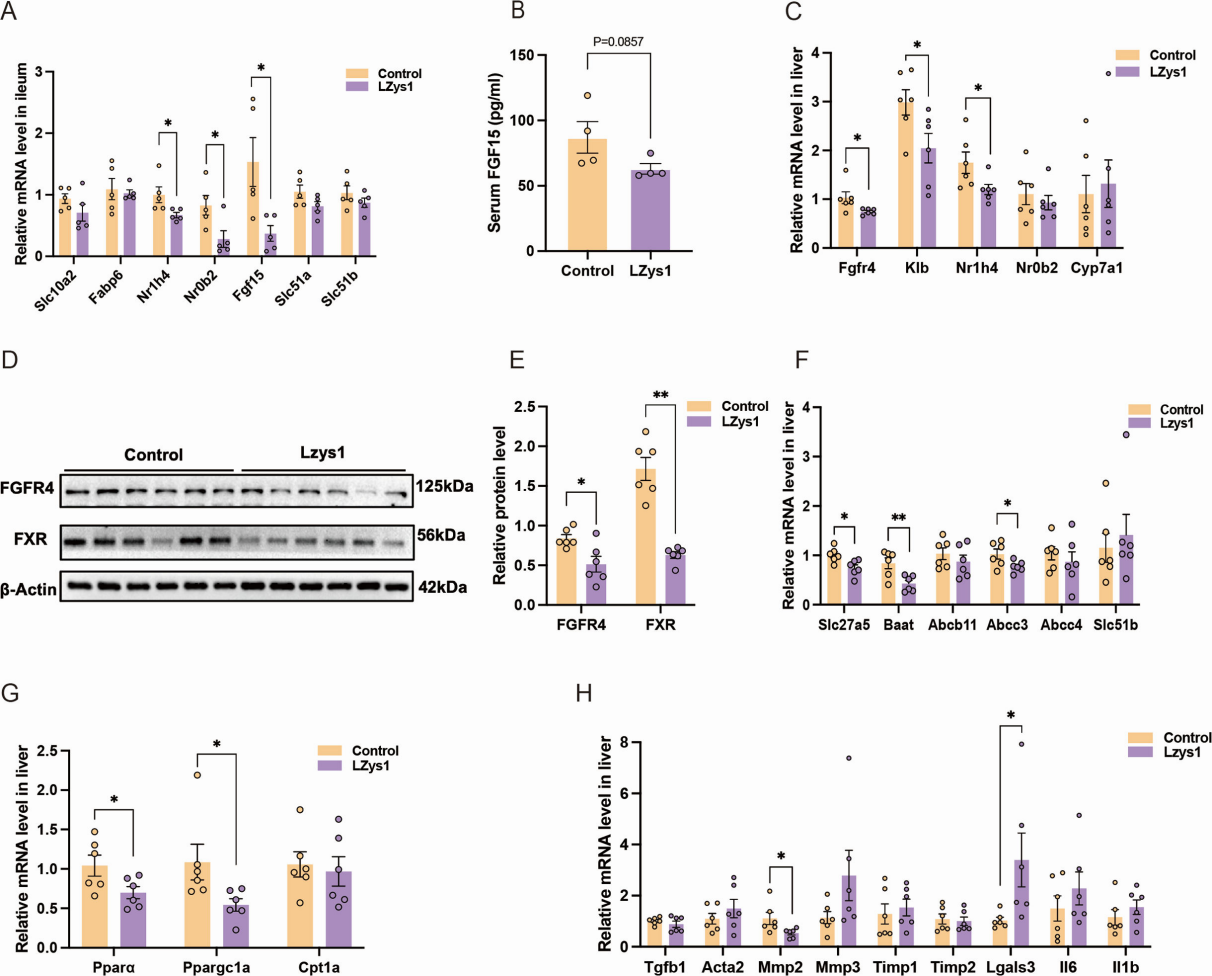
*L. petauri* LZys1 decreases ileal-derived circulating FGF15 and downregulates hepatic FGFR4-FXR-mediated BA metabolism and fatty acid oxidation. (**A**) The expression levels of genes associated with the FXR/FGF15 pathway in the ileum, normalized to β-actin (*n* = 5, per group). (**B**) Serum FGF15 concentration (*n* = 4, per group). (**C**) Expression of genes associated with the FXR/FGF15 pathway in the liver, normalized to β-actin (*n* = 6, per group). (**D**) Representative Western blot images illustrating the level of FGFR4 and FXR protein in the liver (*n* = 6, per group). (**E**) The relative grayscale values of Western blot images in (**D**). (**F**) The expression of genes associated with BA secretion and synthesis of the conjugated BAs in the liver, normalized to β-actin (*n* = 6, per group). (**G**) The expression of genes associated with oxidation of fatty acids in the liver, normalized to β-actin (*n* = 6, per group). (**H**) The expression of profibrotic growth factors and proinflammatory cytokine genes in the liver, normalized to β-actin (*n* = 6, per group). Data (**A–H**) are expressed as mean ± SEM and compared by two-sample *t*-test. *P < 0.05, **P < 0.01.

BAs are the endogenous ligands for the nuclear receptor FXR. Activation of ileal FXR by unconjugated BAs promotes a series of pathways that regulate BA synthesis and transit (28). To determine the molecular mechanism of disorder of BA metabolism after *L. petauri* LZys1 administration, we first assessed the key factors in BA uptake and transition in the ileum. The LZys1 group exhibited relatively decreased expression of ASBT (Slc10a2) and IBABP (Fabp6) gene, responsible for a lower effectivity of BAs retaking from enteric cavity toward intestinal epithelial cells, along with the significant decreasing expression of FXR (Nr1h4), SHP (Nr0b2), and FGF15 (Fgf15). The OSTα (Slc51a) and OSTβ (Slc51b) gene expressions were also reduced to a certain degree (Fig. 5A). Then the serum concentration of FGF15 level was also reduced (Fig. 5B), sending less signal from the ileum to the liver. Consistent with the ileum, the LZys1 group showed scientifically less expression of FGFR4 (Fgfr4), FXR, and KLB (Klb) genes (Fig. 5C). The rate-limiting enzyme CYP7A1, represented for the classic pathway, exhibited a relative increase (Fig. 5C). Likewise, the FGFR4 and FXR protein expressions in liver were also reduced (Fig. 5D and E), representing that *L. petauri* LZys1 administration inhibits BA metabolism through FXR/FGF15/FGFR4 signaling. Having detected a reduction in conjugated BAs, we examined gene expressions of the key enzyme, BACS (Slc27a5) and BAAT (Baat), that form conjugated BAs. As shown in the figure, BACS and BAAT expressions were significantly reduced in the liver (Fig. 5F). The key transporter for BA secretion is also regulated by FXR. The BSEP (Abcb11) responsible for BA secretion to bile duct exhibits relatively reduced gene expression in the LZys1 group. The expression of MRP3 (Abcc4) was significantly reduced; meanwhile, the expression of MRP4 (Abcc4) and OSTβ showed relative reduction (Fig. 5F). FXR is one of the nuclear receptor families; it may activate PPARα to have a beneficial synergistic effect on lipid homeostasis (29). In contrast to the control group, the Lzys1 group exhibited lower expression in Pparα, Ppargc1a, and Cpt1a genes (Fig. 5G), suggesting that decreasing FXR expression leads to a disturbance in lipid metabolism. Additionally, the LZys1 group presented significantly upgraded expressions of inflammatory damage genes and pro-inflammatory cytokine genes like galectin-3 (Lgasl3). The expression of other genes like αSMA (Acta2), IL-6 (Il6), and IL-1β (Il1b) also slightly increased(Fig. 5H).

## DISCUSSION

**Fig 6:**
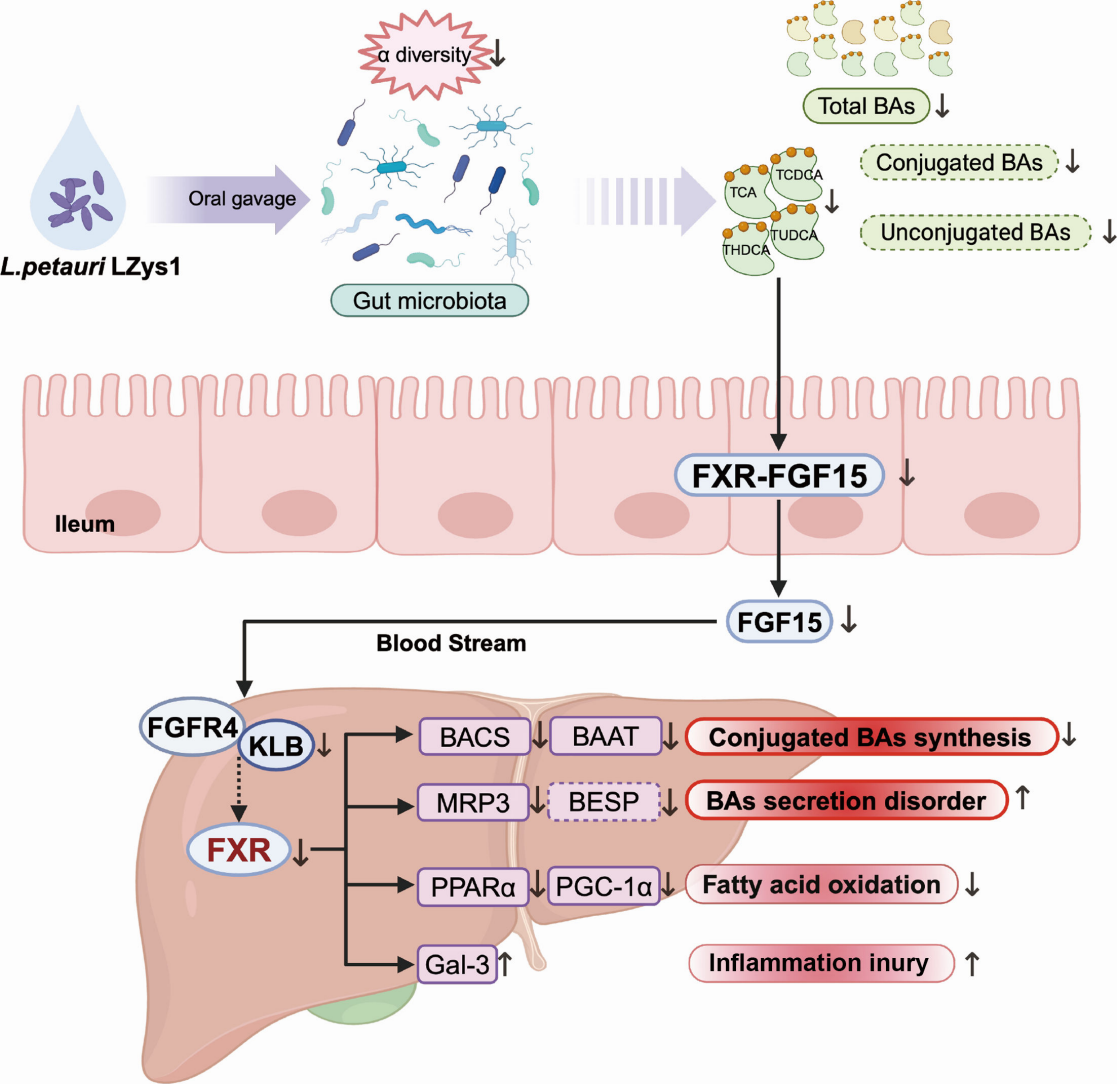
*L. petauri* LZys1 modulates gut microbiota, diminishes ileal FXR-FGF15 signaling, and regulates hepatic function.

In this study, we sought to elucidate the impact of *L. petauri* LZys1 on host intestinal microbiota metabolism and hepatic physiological metabolism. We found that the administration of *L. petauri* LZys1, was associated with liver weight gain and decline of liver function, and suppression of BA profiles. On the basis of these findings, we proposed that the observed changes in liver status are associated with the enterohepatic FXR–FGF15 axis, leading to a decrease in conjugated BA synthesis, BA excretion, and lipid oxidation (Fig. 6G).

The favorable acid production profile of *Lactococcus* and other strains has been exploited for years for the production of fermented foods. These are commonly used as dairy starters in the food industry and have been established to have potential properties (30). Nine genera belong to the genus *Lactococcus*, including *Lactococcus garvieae*, *Lactococcus lactis*, *Lactococcus plantarum*, *Lactococcus formosensis*, *Lactococcus piscium*, *Lactococcus fujiensis*, *Lactococcus chungangensis*, *Lactococcus taiwanensis*, and *Lactococcus raffinolactis*; however, not all recognized species are suitable for the safe production of lactic acid (31, 32). For example, *L. raffinolactis* has been identified as an opportunistic pathogen in fish (33), and *L. garvieae* is an emerging pathogen associated with the consumption of raw fish and seafood and has been implicated in an increasing number of human infections, primarily in endocarditis (34, 35). Isolated from sugar gliders in 2017, *L. petauri* is the most recently discovered and well-described *Lactococcus* species (36), although to date, there has been comparatively little research conducted on this bacterium, with most of these studies focusing on identification and taxonomic analysis. These studies have indicated that phylogenetically, *L. petauri* is closely related to *L. garvieae*, and similar to this species, we speculate that *L. petauri* is also conditionally pathogenic with zoonotic potential. In this study, we found that oral administration of *L. petauri* LZys1 was associated with a disruption of the gut microbiota, suppression of BA metabolism, and adverse effects on liver function, thereby revealing the potentially detrimental effects of this newly isolated species, and thus emphasizing the need for caution in the industrial and medical use of probiotics in the future.

BSH activity is generally observed in gram-positive bacteria, particularly among species in the genera *Clostridium*, *Enterococcus*, *Bifidobacterium*, and *Lactobacillus* (37–39), in which it catalyzes the conversion of conjugated to unconjugated BAs, thereby enhancing BAs’ hydrophobicity and binding to intestinal epithelial cell membranes. Furthermore, the findings of some studies have indicated that BSH activity is thought to be a bacterial adaptation to the toxic effects of conjugated BAs, such as the degradation of cell membrane lipids (40, 41). Similarly, a large body of research evidence has indicated that administration of gut microbiota, such as *Akkermansia* and *Bifidobacterium*, influences the BA metabolism and alters the intestinal microbiota, whereas a reduction in the LZys1 group including species of *Akkermansia* demonstrated to contribute to the regulation of BA profiles via the FXR–FGF15 axis in non-alcoholic fatty liver disease (NAFLD) (42). We have found that the alterations in the gut microbiota are associated with the decrease in the BA content of the ileum and the change in the proportion of conjugated BAs in the liver.

With respect to the alteration in BA profiles, we observed reductions in both the total amounts of BAs and the major conjugated BAs in the ileum and liver. At present, however, there is debate regarding the FXR-activating potentials of BAs. The findings of some studies in which FXR has been overexpressed in human or monkey cell lines have revealed a rank order of stimulatory capacities of the BAs, among which, CDCA has the most pronounced effects, followed by DCA, LCA, and finally CA. However, in mice, this ranking has been found to differ under physiological and non-hepatotoxic conditions in mice (43). In addition, BA concentration has been established to be an important factor in determining FXR activity. Although findings differ in this regard, there is a consensus that the total pool of BAs has the potential to activate FXR (44). In the present study, we observed the significant reductions in the ileal concentrations of TDCA, TωMCA, TβMCA, TCDCA, and TUDCA in the ileum and found that these changes were highly associated with bacterial taxa such as *Eubacterium*, *Ruminococcaceae*, *Lachnospiraceae,* and so on. It has previously been shown that TβMCA, as an initiator of intestinal FXR signaling, promotes the production of hydrophobic BAs, thereby inhibiting the downregulation of FGF15 in mice (45). Moreover, a low concentration of serum FGF15 has been found to inhibit FGFR4/KLB, thereby augmenting the relative expression of CYP7A1 (46). On the basis of the phenotype of suppressed BA flow in the enterohepatic circulation, we propose a different hypothesis. Given that the gut microbiota play key roles in BA metabolism under physiological conditions and, through various modifications, contribute to enhancing the diversity and overall hydrophobicity of the BA pool (6), we suggest that the reduction in BA levels observed in the LZys1-treated mice may result from a disruption in the production of microbial metabolites. More specifically, we speculate that this could be attributable to an enhanced excretion of BAs in the feces. In this regard, the findings of probiotic studies have indicated that the strain *VSL#3* induces BSH activity, thereby promoting BA deconjugation via an inhibition of the FXR–FGF15 axis, which in turn leads to an increase in fecal BA excretion (47), and can probably be ascribed to a reduction in the expression of BA synthetic enzymes.

Among the mechanisms whereby an inhibition of FXR might lead to hepatic toxicity is the disturbance of BA metabolism. Consistent with the present study, mice lacking the FXR, which is involved in maintaining hepatic BA levels, are highly sensitive to cholic acid-induced hepatotoxicity (48). BA secretion across the hepatic canalicular membrane is generally considered to be the rate-limiting step in the BA enterohepatic circulation. BA concentrations in liver and intestine reflect the efficiency of bile duct secretion to a certain extent. In the previous report, drugs known to cause intrahepatic cholestasis, such as cyclosporine A, rifampicin, and glipalamide, inhibit BSEP-mediated taurocholate transport (49). We observed a downregulation of BA transporters in mice. In this regard, it has been found that in humans, a defect in the ABCB11 gene encoding BSEP leads to progressive familial intrahepatic cholestasis-II, which clinically manifests as an elevation in transaminase levels and subsequent development of hepatic failure (50). It has previously been shown that chemical inhibition of BSEP causes minimal canalicular efflux, and that the accumulation of hepatotoxic BA in the hepatocytes causes damage to the mitochondrial membrane (51). Drugs and their metabolites may act as inhibitors to BSEP. Rifampicin, glibenclamide, and ciclosporin acquire forms of intrahepatic cholestasis even as drug-induced liver injury, due to the competitive inhibition of BSEP (52). In this context, by promoting the release of endobiotics and xenobiotics into the blood, the expression of MRP3 and MRP4 in the sinusoidal membrane of hepatocytes is believed to constitute a salvage mechanism under circumstances in which the primary pathway for the elimination of BAs via BSEP is impaired and is responsible for the relief of the endobiotics and xenobiotics into blood. In case of obstructive cholestasis with gallstones, there is an upregulated expression of MRP3, thereby providing evidence to indicate a protective role for MRP3 against the excessive accumulation of BAs (53). In a mouse model of cholesterol gallstones, the activation of FXR-MRP3/MRP4 signaling has been demonstrated to be associated with a significant reduction in lithogenic diet-induced gallstones and hepatic steatosis following the administration of *Limosilactobacillus reuteri* and *Lactiplantibacillus plantarum* (54). Disruption of BA metabolism may also directly modulate liver function. A reduction in the proportion of conjugated BAs leads to a decrease in the efficiency of BA transport, while possibly directly affecting hepatocyte function through the accumulation of unconjugated BAs.

Activation of FXR has also been shown to be associated with a reduction in inflammation in diabetes (55). Previously, Nr1h4-null mice have been observed to be characterized by a full spectrum of NAFLD pathologies, including steatosis, inflammation, fibrosis, and carcinogenesis (56), and a deficiency of Nr1h4 in mice has been established to be linked to increases in the levels of triglycerides and cholesterol, comparable to the lipid and lipoprotein metabolism profiles of FXR (57). Furthermore, in conjunction with Takeda G protein-coupled receptor 5, FXR has been shown to induce human PPARα and PPARγ coactivator protein-1α, the activities of which promote increases in mitochondrial oxidative phosphorylation and energy metabolism, thereby reducing the likelihood of obesity and diabetes (58). Previous studies have also shown that HDCA acts as an inhibitor to alleviate NAFLD by promoting the expression of hepatic CYP7B1, as well as the abundance of probiotic species, such as those in the genus *Parabacteroides* in mice, thereby enhancing lipid catabolism via PPARα signaling (59). In addition, by upregulating the transcription of Cpt1a, Pparα, Mcad, Acsl, and Ppargc1a, it has been demonstrated that fexaramine, an agonist of FXR, contributes to alleviating insulin resistance and lipid accumulation in NAFLD mice (60). Additionally, increased free fatty acids released from adipose tissue are transported to hepatocytes, wherein they are converted to triglycerides, which stimulate the production of FGF21, and in turn reduce serum triglycerides by stimulating energy metabolism and inhibiting mouse mammalian target of rapamycin complex 1 signaling, thereby contributing to a reduction in hepatic damage and steatosis (61).

By regulating leukocyte activation and interacting with the Nrf2 and AMPK pathways, FXR may also play a protective role in the mitochondria and reduce endoplasmic reticulum-associated stress (62). The immunomodulatory effects of FXR may also be mediated via a direct inhibition of an NF-κB-dependent pathway. The NF-κB signaling pathway plays a key role in the production of inflammatory mediators and the mobilization of pro-inflammatory cytokines and enzymes (TNFα, IL-1β, IL-6 ) (63, 64), thereby promoting a continued cycle of inflammation. Among these cytokines, IL-6 activates the Janus kinase–signal transducer and activator of transcription 3 pathway, which initiates the progression of hepatic injury and carcinogenesis (65). Moreover, in non-alcoholic steatohepatitis, a deficiency in FXR has been found to be associated with increases in liver inflammation and fibrosis, and consistent with our findings in this study, those of previous *in vitro* and *in vivo* studies have indicated that FXR inhibits the expression of profibrotic growth factors and pro-inflammatory cytokines in the kidney. In addition, reactive oxygen species (ROS) can directly activate NF-κB and induce direct DNA damage in cells.

The finding of previous studies has also indicated that FXR regulates hypoxia-inducible factors (HIFs), which in turn promote the expression of Galectin-3 (Gal-3) (66). Among these factors, under hypoxic conditions, HIF-2 has been shown to be involved in hypoxia-induced FXR transcriptional repression under hypoxic conditions (67). HIFs also play roles as the main regulators of oxygen balance in the human body and may contribute to hypoxic damage in multiple organ systems, whereas Gal-3 has been established to play roles in a range of pathophysiological processes including organ fibrosis, inflammation, obesity, and even cancer (68).

However, despite our important findings, this study does have certain limitations. Notably, we have yet to establish the precise mechanisms whereby *L. petauri* LZys1 regulates the enterohepatic FXR-FGF15 pathway, metabolism of BAs, and the gut microbiota. In particular, the downstream mechanism underlying the impairment of liver function associated with a repression of FXR requires further in-depth analysis. Though further research is warranted to determine the specific molecular mechanisms underlying the crosstalk between the gut microbiomes and BAs in response to the administration of *L. petauri* LZys1, we would like to advocate the clinical use of probiotic preparations with follow-up of liver function to avoid adverse effects due to imbalance of gut flora and probiotic overdose.

## Data Availability

The complete genome sequence of *L. petauri* LZys1 was uploaded to DDBJ/ENA/GenBank under accession number JABTTD000000000.1.

## SUPPLEMENTAL MATERIAL

Fig. S1 spectrum.01716-24-s0001.docx Original wb images and repeated wb images.

Table S1 spectrum.01716-24-s0002.docx The parameter of UPLC-MS/MS.

ASM does not own the copyrights to Supplemental Material that may be linked to, or accessed through, an article. The authors have granted ASM a non-exclusive, world-wide license to publish the Supplemental Material files. Please contact the corresponding author directly for reuse.
