# Supplementary figures and images for: Lactococcus petauri LZys1 modulates gut microbiota, diminishes ileal FXR-FGF15 signaling, and regulates hepatic function
Source: Microbiol Spectr. 2025 Apr 17;13(6):e01716-24. doi: 10.1128/spectrum.01716-24 (PMC12131734; doi:10.1128/spectrum.01716-24)

supplementary 2. Original wb images and repeated wb images.
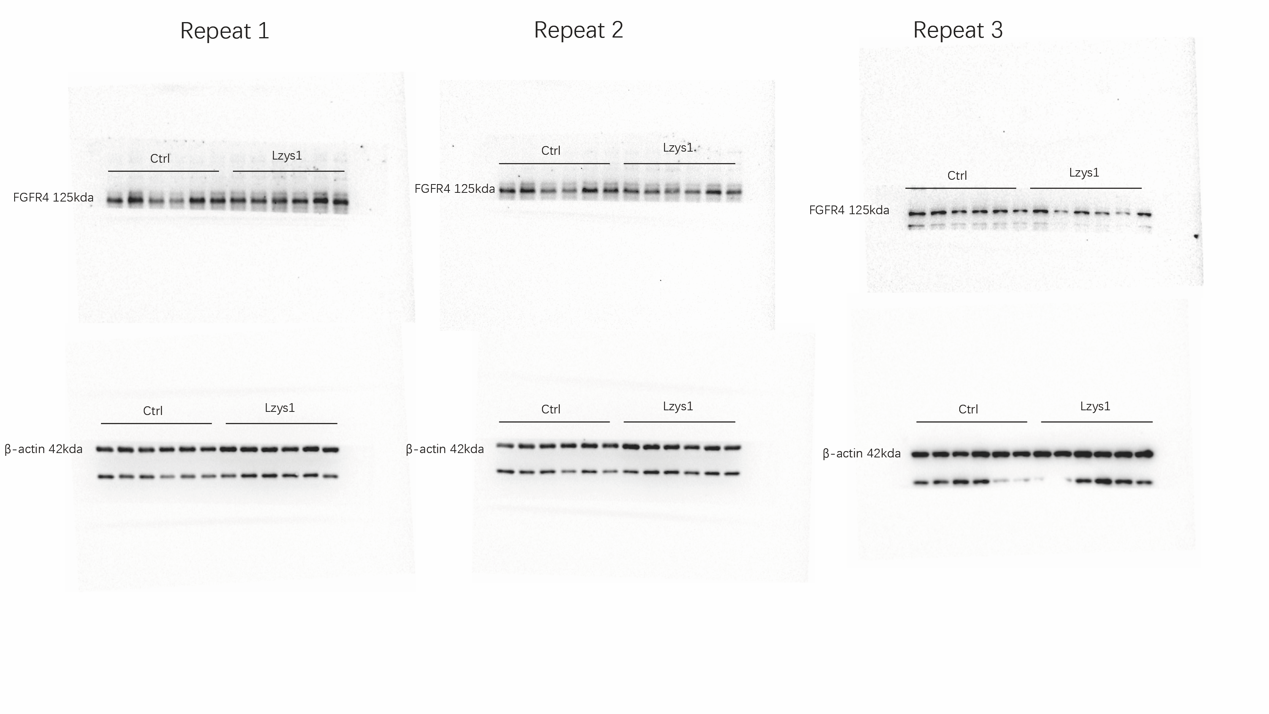

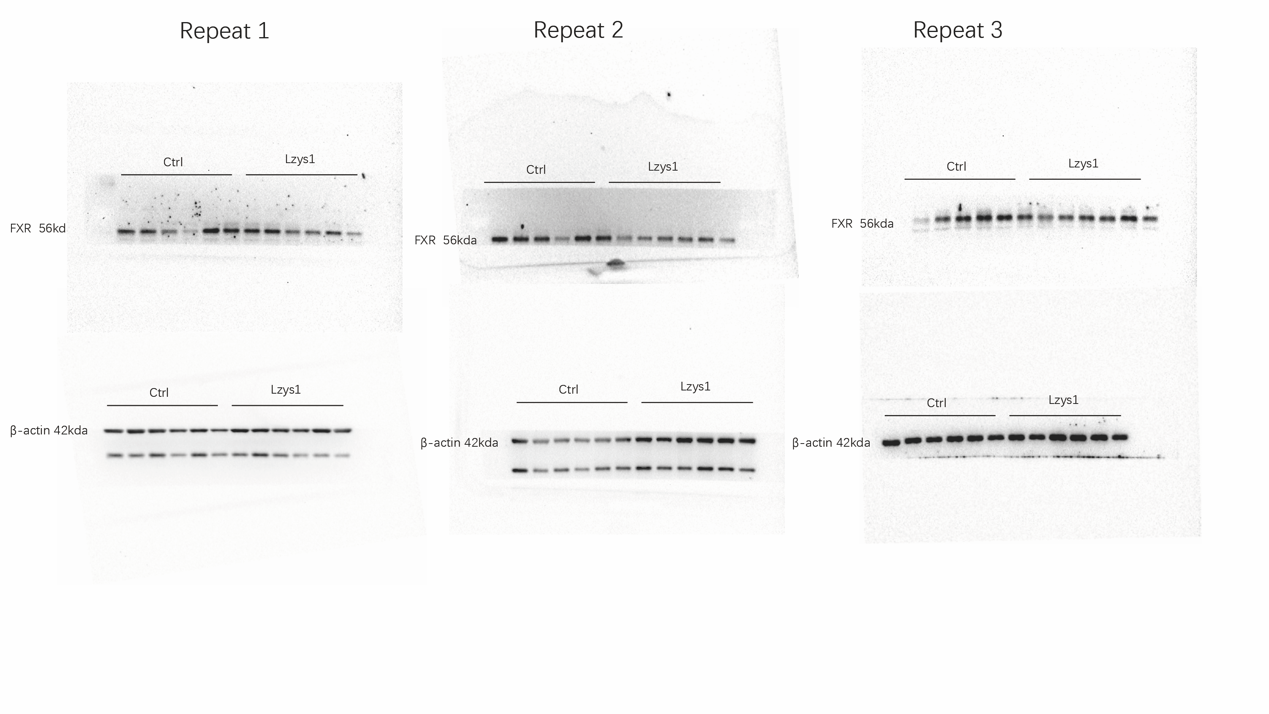

Supplement: Fig. S1 — Original wb images and repeated wb images. [file spectrum.01716-24-s0001.docx]
