# Supplementary material for: Lactococcus petauri LZys1 modulates gut microbiota, diminishes ileal FXR-FGF15 signaling, and regulates hepatic function
Source: Microbiol Spectr. 2025 Apr 17;13(6):e01716-24. doi: 10.1128/spectrum.01716-24 (PMC12131734; doi:10.1128/spectrum.01716-24)
Supplement: Table S1 — The parameter of UPLC-MS/MS. [file spectrum.01716-24-s0002.docx]

Supplementary 1. The parameter of UPLC-MS/MS

| UPLC-MS/MS | Parameter |
| --- | --- |
| Column | ACQUITY UPLC Cortecs C18 1.6 μM VanGuard pre-column（2.1×5 mm）  ACQUITY UPLC Cortecs C18 1.6 μM analytical column（2.1 × 100 mm） |
| Mobile phase | A=10 mM ammonium acetate with 0.25% acetate acid; and B=acetonitrile ：methanol：isopropanol（8:1:1） |
| Gradient elution | 0-0.3 min （5% B），0.3-0.5 min（5-10% B）， 0.5-2 min （10-15% B），2-3 min（15-30%B），3-6 min （30%B），6-8 min （30-35%B），8-9 min （35-40%B），9-10 min（40%B），10-15min（40-75%B），15-15.5min（75-100%B），15.5-16.2min（100%B），16.2-16.3min （100-5%B），16.3-17min （5%B） |
| Flow rate | 0.4 mL/min |
| Capillary voltage | 2.0Kv |
| Temperature | 150℃ |
| Desolventisation temperature | 550℃ |
| Desolventised airflow | 1000L/Hr |
